# Spatial data analysis of malnutrition among children under-five years in Ethiopia

**DOI:** 10.1186/s12874-021-01391-x

**Published:** 2021-10-27

**Authors:** Haile Mekonnen Fenta, Temesgen Zewotir, Essey Kebede Muluneh

**Affiliations:** 1 Department of Statistics, College of Science, Bahir Dar University, Bahir Dar, Ethiopia; 2 Department of Public Health, College of Medicine and Health Sciences, Bahir Dar University, Bahir Dar, Ethiopia; 3 School of Mathematics, Statistics and Computer Science, College of Agriculture Engineering and Science, University of KwaZulu-Natal, Durban, South Africa

**Keywords:** Composite index of anthropometric failure (CIAF), Fixed effects, Geo-additive model, Spatial effects

## Abstract

**Background:**

Childhood malnutrition is a major cause of child mortality under the age of 5 in the sub-Saharan Africa region. This study sought to identify the risk factors and spatial distribution of the composite index of anthropometric failure (CIAF).

**Methods:**

Secondary data from 2000, 2005, 2011, and 2016 Ethiopian Health and Demographic Survey (EDHS) were used. The generalized geo-additive mixed model was adopted via the Integrated Nested Laplace Approximation (INLA) with a binomial family and logit link function.

**Results:**

The CIAF status of children was found to be positively associated with the male gender, the potency of contracting a disease, and multiple births. However, it was negatively associated with family wealth quartiles, parental level of education, place of residence, unemployment status of mothers, improved sanitation, media exposure, and survey years. Moreover, the study revealed significant spatial variations on the level of CIAF among administrative zones.

**Conclusions:**

The generalized geo-additive mixed-effects model results identified gender of the child, presence of comorbidity, size of child at birth, dietary diversity, birth type, place of residence, age of the child, parental level of education, wealth index, sanitation facilities, and media exposure as main drivers of CIAF. The results would help decision-makers to develop and carry out target-oriented programs.

**Supplementary Information:**

The online version contains supplementary material available at 10.1186/s12874-021-01391-x.

## Introduction

A considerable number of evidence from different sources currently indicate that nearly one in every nine children under the age of five around the globe had single or multiple forms of malnutrition, and nearly half of the deaths among the under-five children population (U5C) is due to poor nutrition [1]. Moreover, this prevalence is persistently higher in the Sub-Saharan African (SSA) region including Ethiopia [2]. In Ethiopia, the proportion of underweight (low weight for age) fell from 47.12 to 21% of children, stunting (too short for age) fell from 51.22 to 37%, and wasting (too low weight for height) fell from 10.37 to 7% of children from 2000 to 2019 [3].

Previous studies on the prevalence of undernutrition in Ethiopia had focused on a single conventional anthropometric index of stunting, underweight, wasting [4–9] separately proposed by World Health Organization (WHO) [2]. However, those conventional indices, when used alone, failed to give true estimates of the real impact of childhood malnutrition. The composite index of anthropometric failure (CIAF) might overcome such limitations through an aggregation of different forms of malnutrition measures [4–7, 10].

Looking into the global experience, one can see that countries such as China, India, Bangladesh, Malawi, and others have adopted the CIAF approach to defining their U5C’s nutritional status [11–16]. Yet such national wide studies are missing in most developing nations like Ethiopia. Besides, the majority of the previous studies carried out on nutritional status in this country have mainly focused on different socio-economic, demographic, or health-related covariates, disregarding spatial and nonlinear effects of covariates. Moreover, the studies didn’t target the impact of climate and environmental covariates which is now widely acknowledged to be a threat to food security and nutrition around the globe [17, 18]. As such climate change directly affects crop production and therefore food availability [19]. Rainfall shortfalls occur frequently, while temperature increase from time to time and hence increases the rate of evapotranspiration [20]. These bring drought, ultimately leading to lower crop yields and worsened food security and nutrition for vulnerable populations [21].

Further, those studies had only reported geographical variations of CIAF at higher (country/region) aggregated levels, and zonal level variation is rarely examined. A closer look into the contents of the studies shows that their CIAF data is masked in higher-level geographical aggregates, and had an adverse effect on lower levels (zones in this context). This is inconsistent with the decentralized system of governance in Ethiopia. The zone is administrative levels where operation planning, resource allocation, and implementation of health services are made. Hence identifying the problem of malnutrition and its variation among administrative zones would provide deeper insight into the country’s health priorities for the under-five children population. Particularly, this would help Zonal health departments to make informed decisions and actions in their planning, follow-up, monitoring, and evaluation of health activities at lower levels [22–25]. Addressing the health inequalities is of considerable importance for the country, requiring a major reform that will ensure access to health care services for the poor and disadvantaged groups in the zones. Therefore, the main aim of this study was to identify linear fixed, nonlinear, and spatial effects of covariates on CIAF among under-five children in administrative zones of Ethiopia.

## Materials and methods

**Fig. 1:**
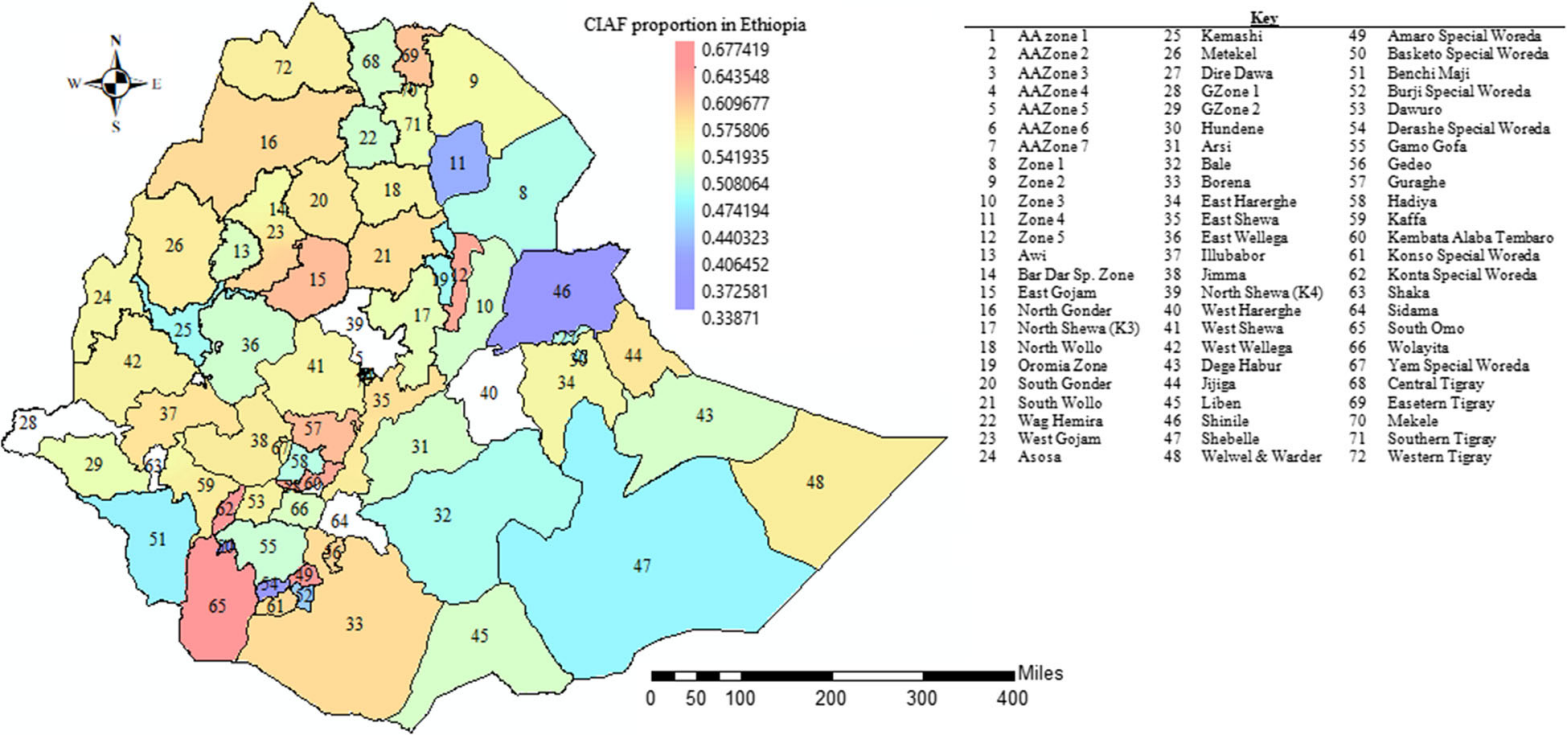
CIAF Prevalence per zones in Ethiopia, based on the EDHS

Ethiopia is the second-largest country in SSA with a population of more than 96 million people, among which more than 13 million are under five. With a surface area of 1.1 million km^2,^ the country shares borders with Eritrea in the north, Djibouti and Somali in the east, Sudan and South Sudan in the west, and Kenya in the south. The government of Ethiopia divides the country into 11 administrative units (regions) including Addis Ababa, the capital city of the country. The regions were further divided into 72 third-level administrative zones mainly based on ethnic groups [26]. Figure 1 shows the spatial prevalence variability of CIAF among the 72 administrative zones of Ethiopia.

### Study data

The dataset of this study consists of 29,792 children from 72 Ethiopian administrative zones over 16 years. We obtained Global Positioning System (GPS) coordinates for 2208 cluster coordinates from EDHS and those clusters were linked with the corresponding administrative III (zones) for spatial analysis. The DHS program randomly displaces cluster coordinates up to 2 and 5 km for urban and rural clusters respectively. This is made to ensure the confidentiality of data and the anonymity of participants. The administrative shapefiles were freely available databases on Global administrative units hosted through the DIVA-GIS project (http://www.diva-gis.org) [27]. This database represents the 72 Ethiopian zones across 11 regions that existed in 2016.

### Variables

**Fig. 2:**
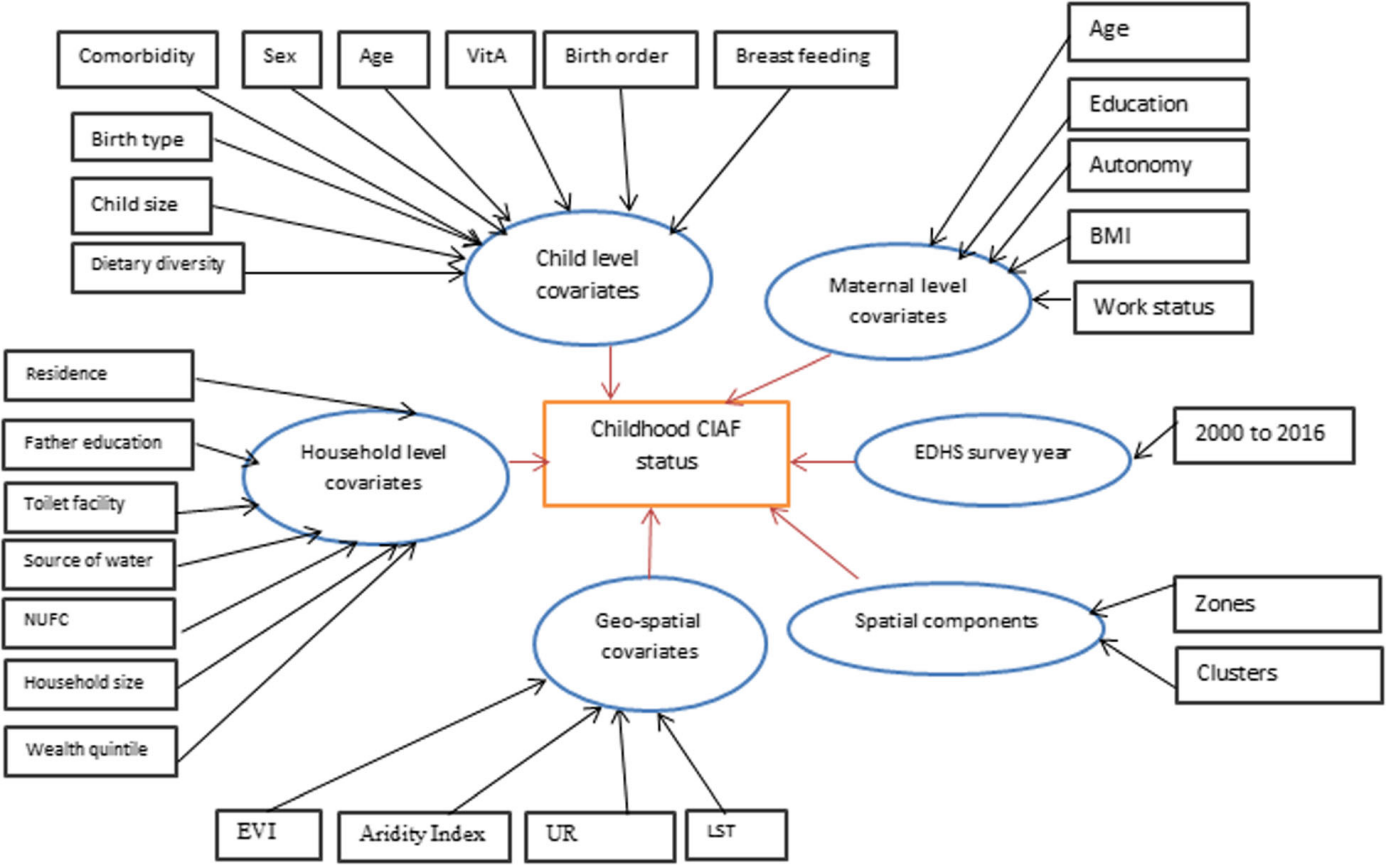
Covariates included in the model

The outcome variable of this study is the composite index for anthropometric failure (CIAF). It is computed by grouping children whose height and weight above the age-specific norm (above − 2 z-scores) and those whose height and weight for their age is below the norm and thus experiencing one or more forms of anthropometric failure. As such, they were classified into seven categories: A-no-anthropometric failure, B-wasting only, C-wasting and underweight, D- wasting, stunting and underweight, E- stunting and underweight, F-stunting only, and Y- underweight only. The CIAF is then calculated by aggregating the six (B-Y) categories [5, 7, 28–30]. The potential risk factors comprise the child, household, and maternal covariates selected based on findings in the literature [8, 31–37] (Fig. 2).

### Geographic covariates

In addition to the child maternal and household DHS covariates, the contextual geographic covariates were obtained from the EDHS. This dataset can easily be linked to the original EDHS datasets through the cluster identifying number (ID) [1, 17, 18, 38–40]. These factors were selected based on previous studies which demonstrated that the nutritional status was correlated with key climate factors [41–45]. The key contextual climate factors in the study include the average number of drought episodes (1, low and 10, high), aridity index defined as the ratio of annual precipitation (0, most arid to 300, most wet), Built-up index (UR) between 0.00 (extremely rural) and 1.00 (extremely urban), Daytime Land Surface Temperature (LST: The average annual land surface temperature during the day), and Enhanced Vegetation Index (EVI).

### Statistical analysis

We adopted three models including the generalized linear mixed model (GLMM), generalized additive mixed model (GAMM), and generalized geo-additive mixed model (GGAMM). The GLMM is a strictly linear regression that assumes a linear effect of the categorical and continuous covariates. Let **W** be an n by p design matrix for p-categorical covariates related to *n* = 29,792 children and which may be linearly associated with the response variable. The response variable Y is assumed to be a member of an exponential family [46] expressed as

1 $${f}_y\left(y;\theta; \varphi \right)=\exp \left\{\frac{y\theta -b\left(\theta \right)}{a\left(\varphi \right)}+c\left(y,\varphi \right)\right\}$$

where a, b, and c are arbitrary functions, *θ* and *φ* is the natural and scale parameter respectively. For the i^th^ child, the GLMM can be expressed as

2 $$g\left\{E\left({Y}_i|\boldsymbol{W}\right)\right\}={\beta}_0+{\sum}_{j=1}^P{\boldsymbol{W}}_{\boldsymbol{ij}}{\boldsymbol{\beta}}_{\boldsymbol{j}}+U{}_{h(i)}$$

Where g(.) is the link function, ***β =*** (*β*_1_, …, *β*_*p*_) is a vector of unknown univariate fixed effect parameters for the categorical covariates and *U*_*h*(*i*)_, *h*_*i*_ =1, 2, . . .,72, is the zone level random effect.

The second model is GAM [47, 48], which is a nonparametric generalized linear model. This model assumes nonlinear functions for the continuous covariates and linear effects of the categorical covariates. Let **X** be the n by q design matrix with the q-dimensional vector of continuous predictors with possible non-linear effects related to *n* = 29,792 under-five children.

3 $$g\left\{E\left({Y}_i|\boldsymbol{W},\boldsymbol{X}\right)\right\}={\eta}_i={\beta}_0+{\sum}_{j=1}^P{\boldsymbol{W}}_{\boldsymbol{ij}}{\boldsymbol{\beta}}_{\boldsymbol{j}}+{\sum}_{l=1}^q{f}_i\left(\boldsymbol{X}\right)+U{}_{h(i)}$$

where ***f*** = (*f*_1_, *f*_2_, …, *f*_*q*_)^*T*^ is a vector of unknown smooth functions.

The third model is GGAMM, which is an extension of GAM by adding a bivariate spatial function. Supposing that the children belong to zone h and cluster k in terms of location, the random spatial effects of the GGAMM is generally given as [49, 50].

$${\phi}_{hk}={f}_{spat}\left({lon}_k,{lat}_k\right)+{U}_h$$

Without loss of generality, the GGAMM model in our study is expressed as;

$${\displaystyle \begin{array}{*{20}l} g\left\{E(Y)\right\}={\beta}_0+{\beta}_1 sex+{\beta}_2 age+{\beta}_3 VA+{\beta}_4 BO+{\beta}_5 BF+{\beta}_6 co+{\beta}_7 sc+{\beta}_8 DDS+\\ {}{\beta}_9 TB+{\beta}_{10} PR+{\beta}_{11} ME+{\beta}_{12} FE+{\beta}_{13} WA+{\beta}_{14} water+{\beta}_{15} toilet+{\beta}_{16} media+{\beta}_{17} WQ+{\beta}_{18} WS+\\ {}{\beta}_{19} year+{f}_1(wet)+{f}_2(tem)+{f}_3(agec)+{f}_4(agem)+{f}_5(evi)+{f}_{spat}\left({lon}_k,{lat}_k\right)+{U}_h\end{array}}$$

The nonlinear term *f*_*spat*_(*lon*_*k*_, *lat*_*k*_) is a function of geographical coordinates of the k^th^ cluster where *lon*_*k*_ *and lat*_*k*_ the longitude and latitude are respectively. The parameter *f*_*spat*_(*lon*_*k*_, *lat*_*k*_) is random effects that capture the unobserved spatial heterogeneity at location *k*, for which some are spatially structured and the others are unstructured. This is denoted as:

4 $${f}_{spat(k)}={f}_{str\left({lon}_k,{lat}_k\right)}+{f}_{unst\left({lon}_k,{lat}_k\right)}$$

### Prior distributions

In the Bayesian context, all the unknown fixed effect parameters and variance (***β,σ***^**2**^) and unknown smooth functions (*f*_*l*_, *l* = 1, 2. . . q, *f*_*str*_ and *f*_*unstr*_) are considered as random variables and this requires specifying the appropriate prior assumptions. Accordingly, for the categorical fixed effect parameters *β* independent diffuse priors, *π* (Φ) ∝ constant are assumed. Also, the non-linear smooth parameters were modeled by second-order random walk prior given by

${f}_l/{f}_{l-1},{f}_{l-2},{\tau}_l^2\sim N\left(2{f}_{l-1}-{f}_{l-2},{\tau}_l^2\right)$ for l = 3, . . .,f with non-informative priors for *f*_1_, *f*_2_ [51–55].

Finally, to capture the unstructured spatial random effects (*f*_*ustr*_), exchangeable normal priors are assumed,${f}_{ustr}\sim N\left(0,{\pi}_{unst}^2\right)$, where ${\pi}_{unst}^2$ is a variance component that allows for over-dispersion and heterogeneity [51–55]. It is important to note at this point, the software R-INLA assign a prior to log (${\pi}_{unst}^2$) which by default is a log-gamma distribution. Yet to model the spatial correlation (structured) components, neighborhoods must be defined among the study zones. Two zones are neighbors if they share common boundaries. The spatial autocorrelation is modeled using a normal distribution with mean zero and a precision matrix that controls correlations between neighbors. Hence, the structured spatial effect of correlations between areas is achieved by incorporating the *f*_*str*_ using the Generalized Markov Random Field (GMRF) priors, where the MRF is defined as [54, 55]

$${f}_{str/{\pi}_{str}^2\Big)}\sim N\Big(0,{\pi}_{str}^2{Q}^{-1}$$) (5)

Such that ${\pi}_{str}^2$ designates the unknown precision parameter that controls the degree of similarities, and Q is the spatial precision matrix that encodes the spatial structure. Hence the (i,j)^th^ element of the spatial precision matrix Q is given by

$$Q= \left\{ \begin{array}{*{20}l} & n_{d} &{}_{d=e} \\ & 1 & d \sim e \\ & 0 & elsewhere\end{array}\right.$$

Where *d*~*e* denotes the area, d is adjacent to e, and n_d_ is the number of adjacent areas to d.

Areas are neighbors if they share common borders [51–55] . Thus, area d with neighboring area e, would have conditional distributions given by

6 $${f}_{str}^d\mid \left\{{f}_{str}^e,d\ne e\right\}\sim N\left(\gamma, \varepsilon \right)$$

where $\gamma =\frac{1}{n_d}\sum_{e\in m}{f}_{str}^d$, $\varepsilon =\frac{\tau_{str}^2}{n_d}$, d and e are adjacent areas in the set of all adjacent areas (*m*) of area s.

Finally, it is important to note that for all unknown variances, the proposed prior distribution is the inverse-Gamma *IG* (*a*_*j*_, *b*_*j*_) [51] where the constant parameters *a*_*j*_ > 0 *and b*_*j*_ > 0 with hyper-parameters *a*_*j*_ = *b*_*j*_ = 0.001 is the standard choice for a weakly informative prior [51–55]. For further illustration, let us assume that Φ presents the vector of unknown parameters in the model and L(.) be the likelihood which is the product of individual likelihoods. With Φ = {*β*, ***σ***^**2**^, *f*_*l*_, *f*_*strc*_}. Under the Bayesian approach, the posterior distribution L(Φ/y) is a function of the prior distribution L(Φ) and the likelihood function L(y/Φ) [52, 54, 56].

### Model diagnostics

In this study, we considered three models mentioned in the methodology part. We used formal criteria like deviance information criteria (DIC) [1, 2] Watanabe_Akaike information criteria (WAIC) [3], and conditional predictive ordinate (CPO) [4, 5] to compare the predictive capability of different competing models including GLMM GAMM and GGAMM. The model with the lowest DIC and WAIC and the highest values of CPO was considered as the best model to fit the data. In the selected model, covariates with an association that was significant at a 10% level were included in the multivariable logit model [49, 50].

The EDHS provided a sample weight for each survey respondent that accounted for the unequal likelihood of selection and non-response. To confirm that our results are representative of the Ethiopian population, the sample weights were included in the analysis through the options pop-up command of “weight” representing the sample weight by “inla” function [57].

### Model validation and evaluation

The test statistic such as the AUC (which is a function of specificity and sensitivity) is used to assess the overall predictive power of the selected model. The AUC measures the ability of the model to correctly predict the presences or absences: specificity measures the percentage of absences correctly predicted, while sensitivity measures the percentage of presences correctly predicted [6–8].

### Sensitivity analysis for priors

Was performed to investigate the influence of the choice of different variance component priors on the final model to check whether the result was unchanged or not. The following specifications were used as recommended: IG (0.001, 0.001), IG (0.01, 0.01), IG (0.5, 0.0005), and (1, 0.026) of different degrees of uncertainty [51, 58–62]. The first two equal prior specifications (a = b) have often been as a standard choice on the variances of random effects [60], the third specifications were suggested by [58, 59], for modeling the precision of the spatial effects in MRF models. The remaining prior choice has been proposed by [59, 61, 62] for capturing residual odds of range (0.5, 2.0). Generally, for the large dataset and well-identified parameters, any of the prior choices have minor different effects on the posterior inferences [51]. For the analysis purpose, the Integrated Nested Laplace Approximation (INLA) software package for R was used, this is due to the time efficiency as compared to MCMC [53, 63, 64].

## Results

**Fig. 3:**
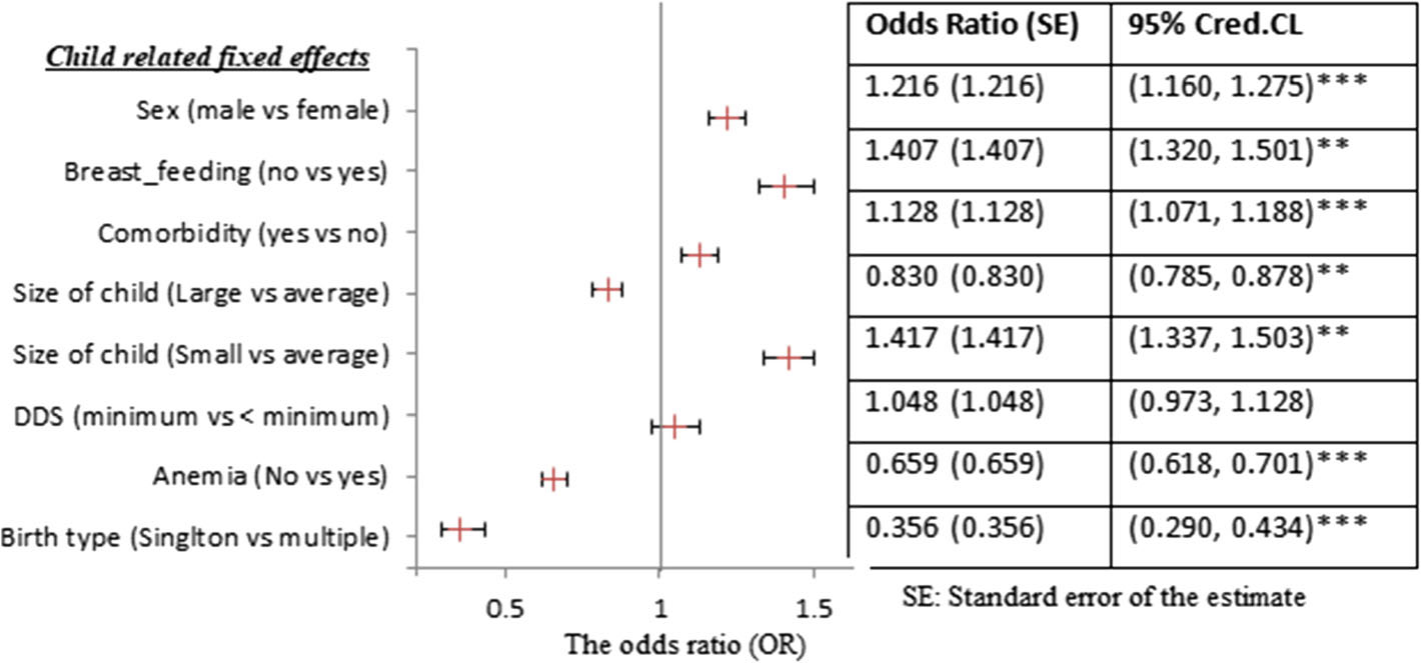
Posterior estimates of the fixed effects of childhood parameters for CIAF among U5C in Ethiopia

This study sought to find out the determinants and spatial patterns of CIAF among U5C using the four cohort DHS studies in Ethiopia. The posterior mean (standard deviation) together with the 95% credible intervals (Crs) is determined for the linear fixed effect parameters included in the model. These figures are comprehensively presented in Figs. 2, 3 below. Also, the positive coefficients corresponding to an increased risk for the CIAF are demonstrated in the table.

### Fixed effects

The findings revealed that the CIAF status of children under five in Ethiopia is positively associated with the male gender. The male under-five children are 22% more likely to be malnourished as compared to female under-five children. Children with comorbidity are 1.407 times more likely affected by malnutrition as compared to those without comorbidity.

The odds ratio of U5C with large/small size at birth decreased /increased by 0.83/1.417 respectively as compared to average size children at birth; the CIAF status of U5C is negatively associated with singleton birth type, i.e. the odds ratio of singleton birth is decreased by 64.4% compared with the multiple birth types (Fig. 3).

**Fig. 4:**
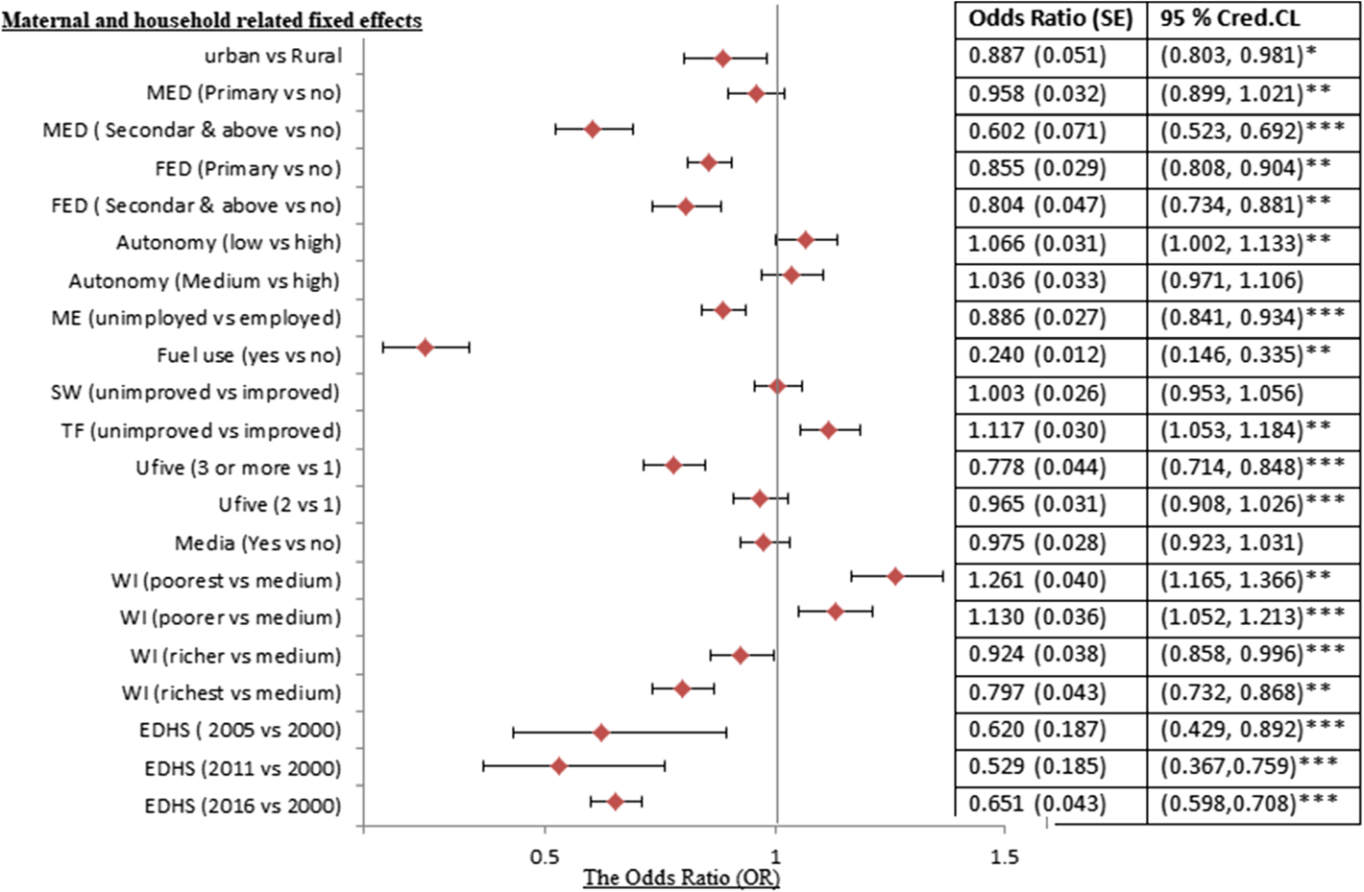
Posterior estimates of the parameters of maternal and household level covariates associated with CIAF among U5C in Ethiopia

Turning to the link between the CIAF status of children and the educational attainments of their parents, one observes a negative association between the two. Those children born from mothers with primary, and secondary and above were 0.958 and 0.602 times less likely affected by malnutrition as compared to those born from mothers with no formal education counterparts. This is markedly different compared to those having no formal education at all. Moreover, those children born from fathers who attained primary, and secondary, and above were 0.855 and 0.804 times less likely affected by malnutrition as compared to their counterparts from those having no formal education. Children born from households with unimproved access to sanitation were 1.116 times more likely affected by CIAF compared with those who got improved sanitation facilities (Fig. 4). Also, children from the low socioeconomic household were, more undernourished than those from high-income backgrounds (the lowest quintile index is positively associated with the CIAF (odds ratio > 1), compared with the middle quintile, the reference group. Particularly, compared against the benchmark category (medium wealth quintiles), children from households with the lowest and lower wealth quintile odds ratio were increased by 26.10 and 13% respectively. In contrast for those from rich and richest wealth quintiles, the odds ratio decreased by 0.924 and 0.797 compared to the baseline category. Finally, the CIAF status of U5C in Ethiopia is negatively associated with the EDHS survey years. As such the odds ratio of CIAF was decreased by 0.620, 0.529, and 0.651 for children whose information has been collected in 2005, 2011, and 2016 respectively compared to the 2000 group, the reference category in the study (Fig. 4).

### Nonlinear effects

**Fig. 5:**
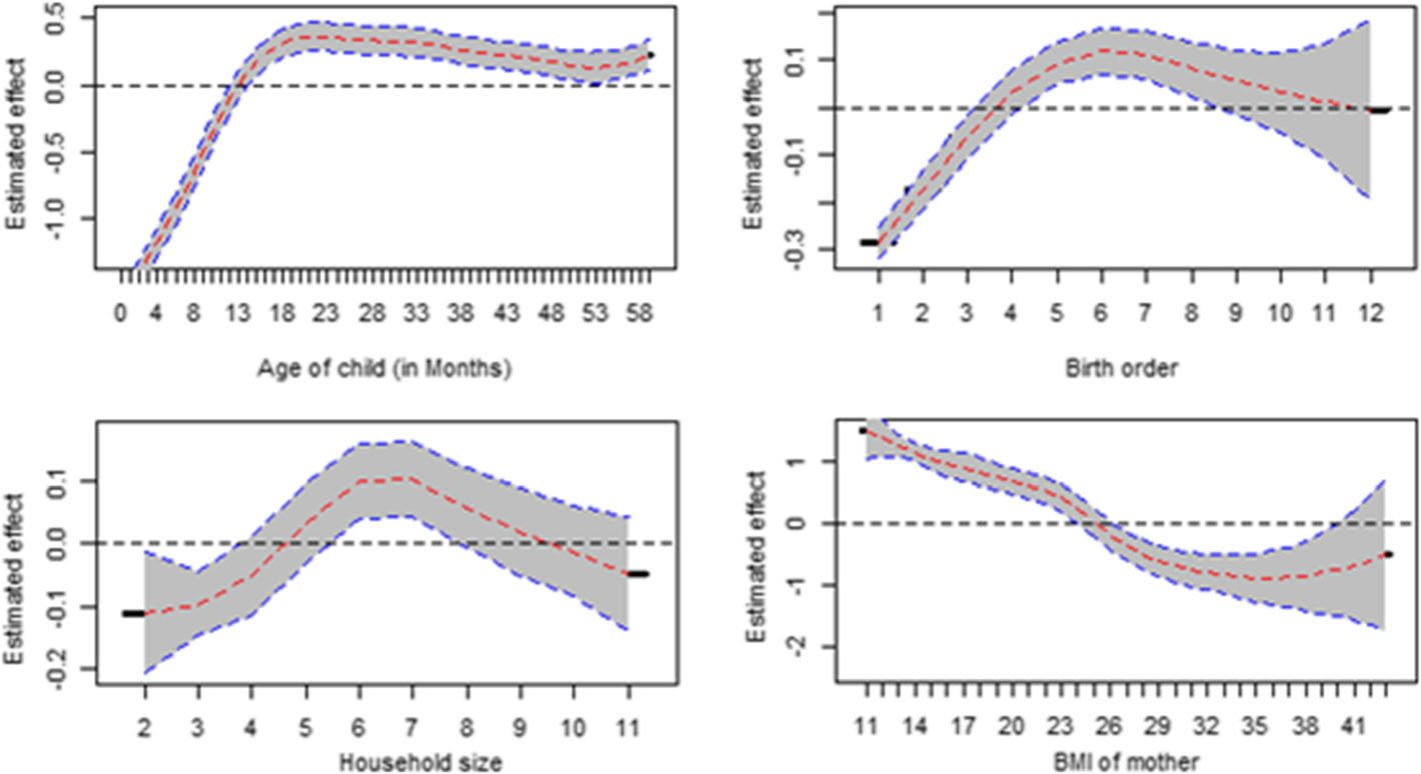
Posterior means of the non-parametric effects of age of the child, birth order, household size and BMI of the mother

**Fig. 6:**
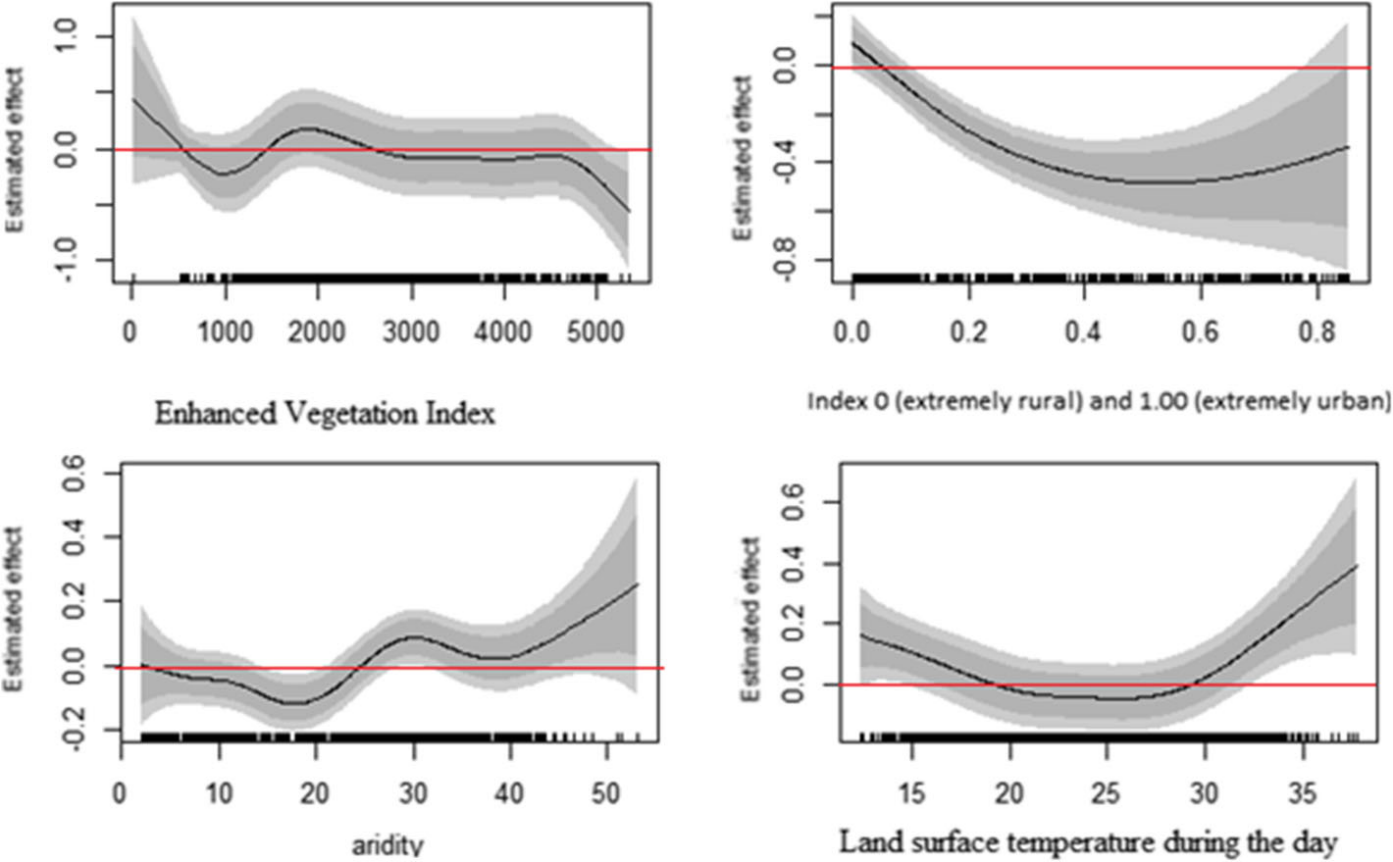
Posterior means of the non-parametric effects of the geo-spatial covariates on child’s CIAF status

The results for the non-linear effects of the continuous covariates and the log-odds of CIAF after accounting for other variables with a 95% confidence interval were presented in Fig. 5 and Fig. 6. The estimates for most of the continuous variables were different from zero, suggesting that there are nonlinear effects (Table 1).

**Table 1:** Regression coefficient and variance component estimation of the nonlinear terms

	Estimate	SE	LL	UL
Child’s age	0.213	0.0370	0.140	0.2856*
Child’s birth order	0.0045	0.0057	0.000	0.0156
Family size	0.0016	0.0021	0.000	0.0058
BMI of mother	0.0012	0.0071	0.000	0.0152
Enhanced vegetation index	0.07	0.0123	0.046	0.0942*
UR index	0.013	0.0012	0.011	0.0153*
Aridity	0.002	0.0154	0.000	0.0321
Land surface temperature	0.005	0.0274	0.000	0.0588
Zone-level random effect	0.056	0.016	0.025	0.087*
Cluster level spatial effect	0.220	0.049	0.123	0.317*

*Keys:* *SE* standard error of the estimate; *LL* Lower Level; *UL* Upper Level; (*) statistically significant at α=0.05

Accordingly, the log-odds of U5C having CIAF is directly but nonlinearly correlated with the age of the child (in months). This shows that the CIAF status of children is negatively associated with the lower age group (up to about 12 months). The influence of a child’s age on its CIAF is considerably high between the age of 15 and the age of 24 months and almost gets static up to the age of about 52 months. The log-odds of CIAF status of U5C were negatively associated with the lower birth orders (birth order < 4). It increases between the birth orders of 4 to 6 and stabilizes at the same level thereafter. Looking into the mother’s body mass index and its impact on the level of their child’s log-odds of CIAF, one can observe the influence expressed in the form of a u-shape in the graph. As such, the result revealed that there is a positive association between the thinness condition of the mother and the CIAF status of their U5C (Fig. 5).

Turning to the last determinant under this category, we observe that the CIAF status of U5C is indirectly but non-linearly correlated with enhanced vegetation index (EVI) and urban-rural (UR) settlement ratios. Particularly, the risk of CIAF was found to be significantly high in the lower EVI and UR. Further, looking into the aridity and land surface temperature (LST) and their impact on the log-odds level of their child’s CIAF there is a negative association between the lower values of the aridity and lst., The influence is depicted in the graphs which take a clear-cut u-shape forms. Finally, while a negative association is evident in the graphs, the log-odds of CIAF increase with an increase in aridity (> 20) and lst (> 30) (Fig. 6).

### Spatial effects

**Fig. 7:**
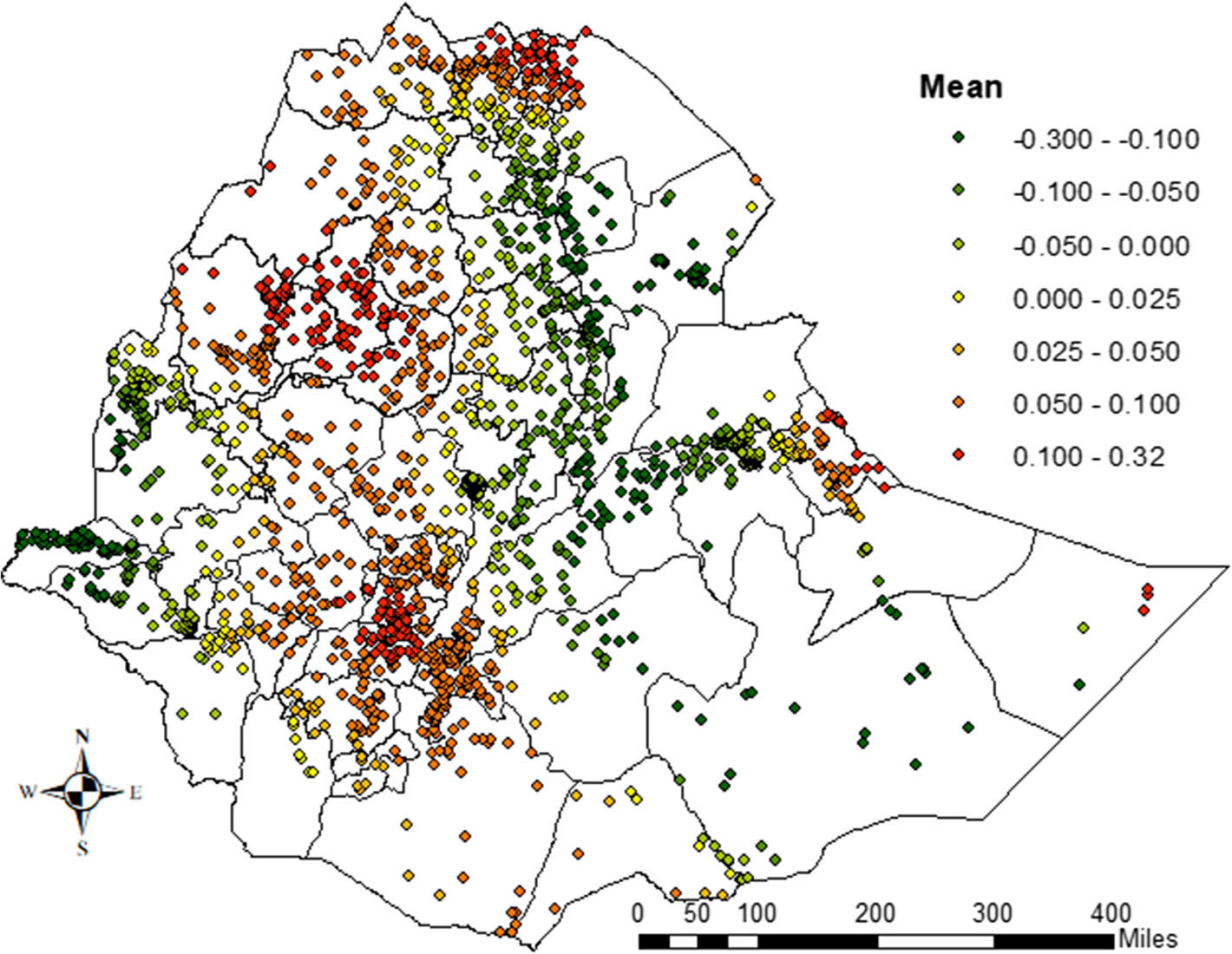
Spatial (structured) variation of CIAF posterior estimates among U5C in Ethiopia

The spatial effects presented in Figs. 6, 7 are based on the GGAMM. It shows both positive and negative spatial effects on the log-odds of CIAF status among the under-five children in Ethiopia. The estimated cluster (structured) and zonal level (unstructured) estimates were generated for 2208 clusters and 72 Ethiopian third-level administrative zones respectively. Moreover, the variance estimates for the zone-level random effect and the cluster-level spatial effects (which account for the spatial autocorrelation) were nonzero and statistically significant (Table 1).

The colors on the map show the log-odds scale, which reveals each administrative zone’s contribution to the odds of a child’s CIAF in U5C.

**Fig. 8:**
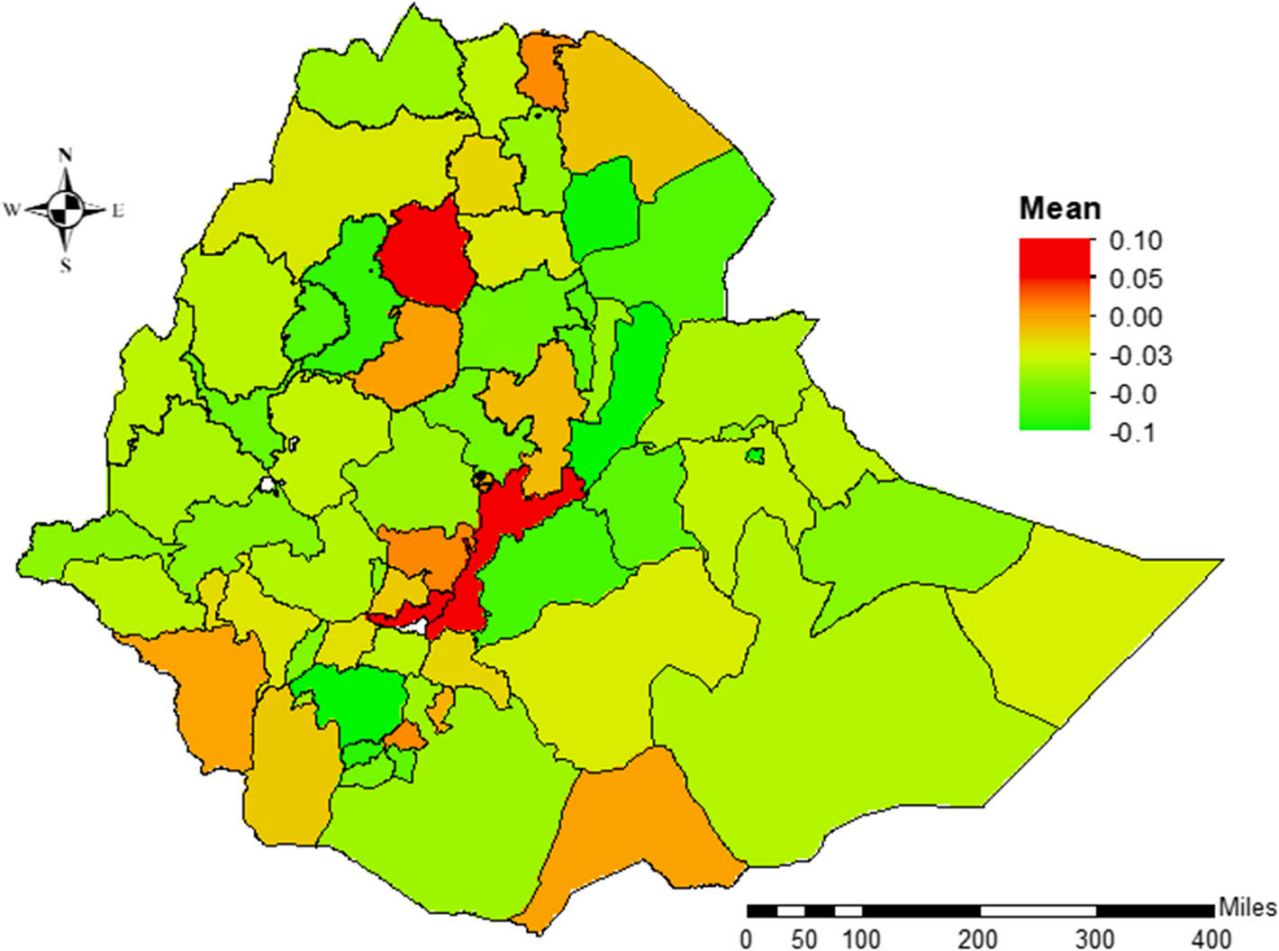
Spatial (unstructured) variation of CIAF posterior estimates among U5C in Ethiopia

The colors ranged from green to red such that the green dot denoting a negative effect on the posterior parameters for the log-odds of CIAF, while the yellow and red dots show the positive effects which are associated with an increased risk of the log-odds of childhood malnutrition. Moreover, the map revealed that the presence of variation in CIAF among under-five children in Ethiopian clusters. Mainly clusters in the northern, north-western, and some parts of the western areas were more likely to be suffered from malnutrition (Fig. 7). The results of the random effects presented in Fig. 8 above reveal the variation across the posterior parameters of CIAF among under-five children at smaller spatial units in administrative zones across the country.

The positive posterior values further reveal the increased log-odds of CIAF in the zones, but the negative values showed the decreased log-odds of CIAF. Notably, children from the zones including the eastern part of Tigray, Gondar, Waghmira, Guraghe, and Alaba Tembaro were more likely to have suffered from malnutrition. Yet most of the subjects from Oromia, Somali, Afar, SNNP regions, and Addis Ababa city were less likely affected. Moreover, the maps of the 95% CrI and SD indicate higher uncertainties about the CIAF distribution in Ethiopia.

#### Model goodness of fit and diagnostics

**Fig. 9:**
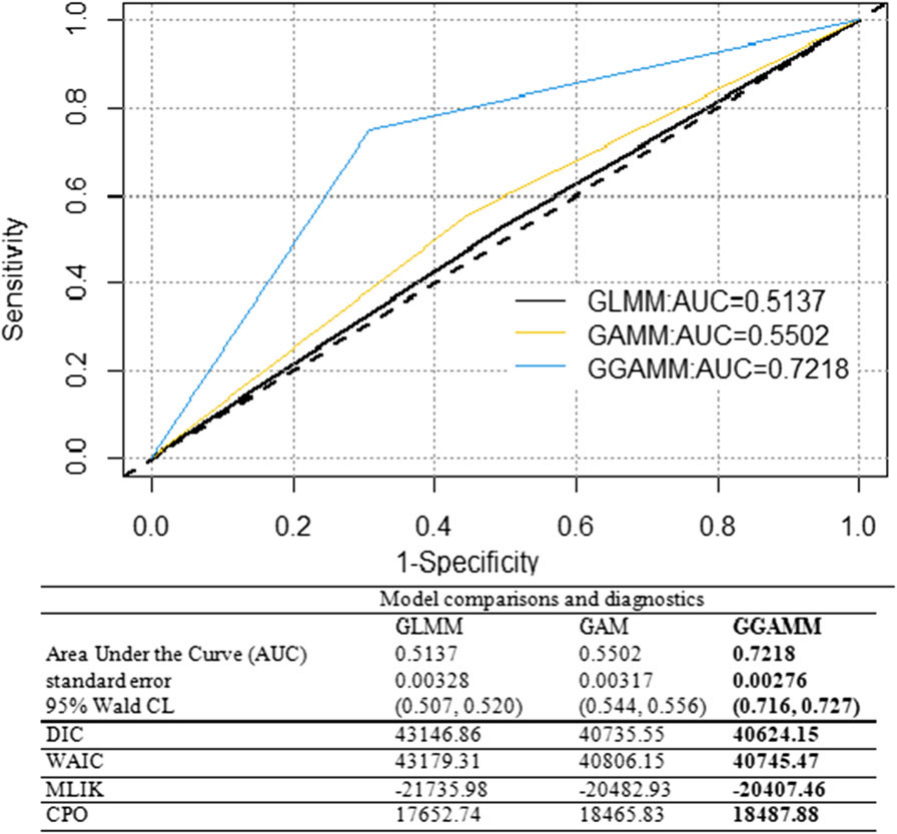
Model diagnostics and goodness of fit

The area under the curve (AUC) for the given three models compared with that of the diagonal line that is always 0.500 (half of the graph) is computed. The GGAMM model has the lowest DIC and WAIC, but the highest values of CPO and AUC indicating that it is the best model for identifying the associated effects of child-household level covariates on CIAF (Fig. 9).

#### Sensitivity analysis

The results are robust to changes in the prior information. It shows that all the four distributions provided almost equivalent values for the parameters and therefore, our model is less sensitive to the choice of the hyper-parameters. Hence, the standard choice of a = b = 0.001 which is the default for R-INLA was implemented to explore this study data with GGAMM (Table 2)

**Table 2:** Summary of the sensitivity analysis of the choice of hyper-parameters with posterior mean-variance (standard error), 95% CI based on GGAMM

spatial effects	a = b = 0.001	a = 0.01,b = 0.01	a = 0.05,b = 0.0005	a = 1,b = 0.026
${\pi}_{str}^2$	0.220 (0.049)	0.229 (0.046)	0.193 (0.055)	0.201 (0.056)
2.5–97.5%	(0.123, 0.317)	(0.139,0.319)	(0.085, 0.301)	(0.091,0.311)
${\pi}_{unstr}^2$	0.056 (0.016)	0.058 (0.015)	0.055 (0.011)	0.055 (0.011)
2.5–97.5%	(0.025, 0.087)	(0.028,0.088)	(0.034,0.076)	(0.033,0.077)
DIC	36,597.81	36,596.98	36,598.83	36,598.30

## Discussion

The focus of this study was identifying factors associated with CIAF among U5C and mapping the possible spatial pattern of CIAF at zonal level heath service institutional structures. As such, it is the first study in the country that provides the new measures for the prevalence of malnutrition by considering the aggregated indicators of anthropometric failure and applies them to larger nationally representative datasets in Ethiopia over time.

The major findings of the study demonstrated that, for all EDHS datasets, the CIAF is significantly higher compared to the prevalence reported by conventional indices of stunting, underweight, and wasting [65–67].

The linear fixed effects have demonstrated a significant positive association between the outcome variable (CIAF) and the demographic characteristics of the child’s maternal level of education, and socio-economic level of households. The results, consistent with the studies reported among the SSA [68] regions, revealed that boys are more likely to have CIAF compared to girls. This may be because the morbidity pattern of male children under the age of 5 is more prevalent than female counterparts [68]. Our study further found out that odds of CIAF among children from a rural area are more prevalent compared to those who live in urban areas. This might be because the rural areas are less developed in healthcare facilities and communities lack awareness of food intake.

A further look into the results shows that children from the lowest socio-economic households are more likely to experience CIAF compared to children from the richest households. This is also consistent with previous studies conducted in other developing countries [14, 34, 38, 69–73]. This might be because in rural areas and in the poorest wealth index families; the access to healthcare facilities and a balanced diet is limited which would lead to inadequate nutrient intakes. Moreover, parents with a limited source of income are unable to purchase food and health care services for their children, which, in effect, contribute to undernourishment.

Finally, the CIAF status is found to have negatively and significantly associated with the educational attainments of parents. The evidence also supports the evidence reported by several studies investigating child nutrition [70, 73–76]. The results suggest that the educated parents have a better knowledge of a balanced diet and the health of their children. Strengthening this implication, the risk of malnourishment (CIAF) is almost 43.8% lower for children whose mother attains secondary or higher education than fathers with similar educational attainments. Of course, it is also important to consider the employment status of the mothers in this respect. With more than 63% of women in Ethiopia being unemployed and usually spend more of their time with their children compared to their husbands, they would afford to provide childcare and nourishment services than the fathers could do. Thus, while the level of education significantly contributes to the mothers’ capacity for child care and nourishment, the time factor might have complemented this impact.

Turning to another determinant, the CIAF status of under-five children was negatively associated with improved sanitation facilities. This might be due to the fact that households with improved sanitation reduce the exposure to additional diseases hence decreases the undernutrition of children. However, the use of improved/unimproved water has no significant effects on the CIAF status of children. This might be connected to the fact that in Ethiopia, only 38% of households are using improved sanitation, but more than 61.5% of inhabitants can use improved water.

Apart from socio-demographic determinants the mothers’ access to mass media use also impacted the CIAF status of the children. As such, a significant negative association is observed between the two variables, suggesting that women who are exposed to media learn about the diet and health of their children and allocating even the limited income to devote to the health nutrition of their children. This finding is consistent with previous research evidence reported in other countries [9, 70, 75, 77, 78]. Further, the CIAF status was negatively associated with the DHS year in Ethiopia. Particularly, the risk of malnourishment (CIAF) is almost 10.9, 31, and 39.5% less likely to be malnourished respectively in 2005, 2011, and 2016 as compared to the reference category (the 2000 group). This decline in under-nutrition prevalence over the past two decades evidenced that Ethiopia continued to address childhood malnutrition.

Turning to another evidence in the data, the nonlinear relationship between the age of the child (in months) and CIAF prevalence was similar to that of studies conducted elsewhere [72, 73]. Moreover, children in the youngest age group (in months) had significantly lower CIAF odds than the older age groups. This result is also consistent with different studies [14, 72–74]. This may be because breastfeeding practices common among mothers who spent much of their time with their children [79]. Moreover, the highest CIAF risk was observed in the age group of almost 18 to 31 months and this is because in this age group mothers do not produce sufficient breast milk to fulfill the required nutrition for their children.

Finally, the predicted values from the GGAMM revealed that child under-nutrition (CIAF) remains high in almost all parts of the country with a limited exception that Zones in Addis Ababa, Gambella, Somali, and central parts of the Oromia region showed a lower risk of CIAF. This limited variation could be due to the relatively better health facilities available in these zones.

## Conclusion

The wide-ranging analyses made in the last sections of the paper demonstrate that the study, as the first of its type in using the GGAMM approach in Ethiopia, has successfully modeled the CIAF at the zonal level by using both the EDHS and geospatial covariates. Through this comprehensive modeling, it has found out that child sex, presence of comorbidity, size of child at birth, dietary diversity, birth type, place of residence, child age, parental educational level, wealth index, sanitation facilities, media exposure of mothers and the survey years have direct links with the risk of under-nutrition among children under the age of five in Ethiopia. Moreover, the minimum body mass index, the higher birth order, and the lower urban-rural ratio were associated with the rising of CIAF. Still, as a major contribution to the inquiry on the subject, this study explored and depicted the spatial maps that revealed the areas at the high (low) estimated risk of undernutrition (CIAF) among under-five children in Ethiopian administrative zones. Finally, the generated estimated values of CIAF and the identified factors would provide essential information that helps the decision-makers to allocate limited resources and program implementation in the zones that need more attention in Ethiopia.

## Supplementary Information

**Additional file 1.**

## Data Availability

The dataset used and analyzed during the current study is available from the corresponding author on reasonable request.

## References

[1] FAO, IFAD, UNICEF, WFP, & WHO. The state of food security and nutrition in the world 2018. Building climate resilience for food security and nutrition. Rome: FAO; 2018.

[2] De Onis M (2012). Worldwide implementation of the WHO child growth standards. Public Health Nutr.

[3] Institute, E.P.H. and ICF (2019). Ethiopia mini demographic and health survey 2019: key indicators.

[4] Al-Sadeeq AH, Bukair AZ, Al-Saqladi A-WM (2018). Assessment of undernutrition using Composite Index of Anthropometric Failure among children aged< 5 years in rural Yemen. East Mediterr Health J.

[5] Nandy S, Svedberg P. The composite index of anthropometric failure (CIAF): an alternative Indicator for malnutrition in young children. In: Preedy V, editor. Handb anthropometry. New York: Springer; 2012.

[6] Svedberg P. Poverty and undernutrition: theory, measurement, and policy. Oxford: Oxford University Press for UNU-WIDER; 2000.

[7] Rasheed W, Jeyakumar A (2018). Magnitude and severity of anthropometric failure among children under two years using composite index of anthropometric failure (CIAF) and WHO standards. Int J Pediatr Adolesc Med.

[8] Habyarimana F, Zewotir T, Ramroop S (2014). A proportional odds model with complex sampling design to identify key determinants of malnutrition of children under five years in Rwanda. Mediterr J Soc Sci.

[9] Kassie GW, Workie DL (2019). Exploring the association of anthropometric indicators for under-five children in Ethiopia. BMC Public Health.

[10] Biswas S, Giri SP, Bose K (2018). Assessment of nutritional status by composite index of anthropometric failure (CIAF): a study among preschool children of Sagar block, south 24 Parganas District, West Bengal, India. Anthropol Rev.

[11] Nandy S, Irving M, Gordon D, Subramanian SV, Smith GD (2005). Poverty, child undernutrition and morbidity: new evidence from India. Bull World Health Organ.

[12] Nandy S, Miranda JJ (2008). Overlooking undernutrition? Using a composite index of anthropometric failure to assess how underweight misses and misleads the assessment of undernutrition in young children. Soc Sci Med.

[13] Pei L, Ren L, Yan H (2014). A survey of undernutrition in children under three years of age in rural Western China. BMC Public Health.

[14] Islam MS, Biswas T (2020). Prevalence and correlates of the composite index of anthropometric failure among children under 5 years old in Bangladesh. Matern Child Nutr.

[15] Bejarano IF, Oyhenart EE, Torres MF, Cesani MF, Garraza M, Navazo B, Zonta ML, Luis MA, Quintero FA, Dipierri JE, Alfaro E, Román EM, Carrillo R, Dahinten S, Lomaglio DB, Menecier N, Marrodán MD (2019). Extended composite index of anthropometric failure in Argentinean preschool and school children. Public Health Nutr.

[16] Ziba M, Kalimbira AA, Kalumikiza Z (2018). Estimated burden of aggregate anthropometric failure among Malawian children. South Afr J Clin Nutr.

[17] Smith KR, Woodward A, Campbell-Lendrum C, Chadee DC, Honda Y, Liu Q, Olwoch JM, Revich B, Sauerborn R. Human health: impacts, adaptation, and co-benefits. In: Climate Change 2014: Impacts, Adaptation, and Vulnerability. Part A: global and sectoral aspects. Contribution of Working Group II to the fifth assessment report of the Intergovernmental Panel on Climate Change. [Field, C.B., V.R. Barros, D.J. Dokken, K.J. Mach, M.D. Mastrandrea, T.E. Bilir, M. Chatterjee, K.L. Ebi, Y.O. Estrada, R.C. Genova, B. Girma, E.S. Kissel, A.N. Levy, S. MacCracken, P.R. Mastrandrea, and L.L. White (eds.)]. Cambridge: Cambridge University Press; 2014. pp. 709–754.

[18] Organization, W.H (2014). Quantitative risk assessment of the effects of climate change on selected causes of death, 2030s and 2050s.

[19] Schlenker W, Lobell DB (2010). Robust negative impacts of climate change on African agriculture. Environ Res Lett.

[20] Milly PC, Dunne KA (2016). Potential evapotranspiration and continental drying. Nat Clim Chang.

[21] Wheeler T, Von Braun J (2013). Climate change impacts on global food security. Science.

[22] Gebreyesus SH, Mariam DH, Woldehanna T, Lindtjørn B (2016). Local spatial clustering of stunting and wasting among children under the age of 5 years: implications for intervention strategies. Public Health Nutr.

[23] Collaborators, G.R.F (2016). Global, regional, and national comparative risk assessment of 79 behavioural, environmental and occupational, and metabolic risks or clusters of risks, 1990–2015: a systematic analysis for the Global Burden of Disease Study 2015. Lancet.

[24] Corsi DJ, Chow CK, Lear SA, Rahman MO, Subramanian SV, Teo KK (2011). Shared environments: a multilevel analysis of community context and child nutritional status in Bangladesh. Public Health Nutr.

[25] Griffiths P, Madise N, Whitworth A, Matthews Z (2004). A tale of two continents: a multilevel comparison of the determinants of child nutritional status from selected African and Indian regions. Health Place.

[26] Fetene N, Linnander E, Fekadu B, Alemu H, Omer H, Canavan M, Smith J, Berman P, Bradley E (2016). The Ethiopian health extension program and variation in health systems performance: what matters?. PLoS One.

[27] ESRI, ArcGIS Desktop: Release 10. Redlands: Environmental Systems Research Institute; 2011.

[28] Shit S, Taraphdar P, Mukhopadhyay DK, Sinhababu A, Biswas AB (2012). Assessment of nutritional status by composite index for anthropometric failure: a study among slum children in Bankura, West Bengal. Indian J Public Health.

[29] Mandal G, Bose K (2009). Assessment of overall prevalence of undernutrition using composite index of anthropometric failure (CIAF) among preschool children of West Bengal, India.

[30] Sen J, Mondal N (2012). Socio-economic and demographic factors affecting the composite index of anthropometric failure (CIAF). Ann Hum Biol.

[31] Chowdhury MRK (2016). Risk factors for child malnutrition in Bangladesh: a multilevel analysis of a nationwide population-based survey. J Pediatr.

[32] Adekanmbi VT, Kayode GA, Uthman OA (2013). Individual and contextual factors associated with childhood stunting in Nigeria: a multilevel analysis. Matern Child Nutr.

[33] Aheto JMK, Keegan TJ, Taylor BM, Diggle PJ (2015). Childhood malnutrition and its determinants among under-five children in G hana. Paediatr Perinat Epidemiol.

[34] Bain LE, Awah PK, Geraldine N, Kindong NP, Sigal Y, Bernard N, et al. Malnutrition in Sub–Saharan Africa: burden, causes and prospects. Pan Afr Med J. 2013;15(1). 10.11604/pamj.2013.15.120.2535. 10.11604/pamj.2013.15.120.2535 PMC3830470 24255726

[35] Black RE, Allen LH, Bhutta ZA, Caulfield LE, de Onis M, Ezzati M, Mathers C, Rivera J (2008). Maternal and child undernutrition: global and regional exposures and health consequences. Lancet.

[36] Degarege D, Degarege A, Animut A (2015). Undernutrition and associated risk factors among school age children in Addis Ababa, Ethiopia. BMC Public Health.

[37] Fenta HM, Workie DL, Zike DT, Taye BW, Swain PK (2020). Determinants of stunting among under-five years children in Ethiopia from the 2016 Ethiopia demographic and health survey: application of ordinal logistic regression model using complex sampling designs. Clin Epidemiol Global Health.

[38] Balk D, Storeygard A, Levy M, Gaskell J, Sharma M, Flor R (2005). Child hunger in the developing world: an analysis of environmental and social correlates. Food Policy.

[39] Nkurunziza H, Gebhardt A, Pilz J (2010). Bayesian modelling of the effect of climate on malaria in Burundi. Malar J.

[40] Mayala B (2018). The DHS program geospatial covariate datasets manual.

[41] Alegana VA, Atkinson PM, Pezzulo C, Sorichetta A, Weiss D, Bird T, Erbach-Schoenberg E, Tatem AJ (2015). Fine resolution mapping of population age-structures for health and development applications. J R Soc Interface.

[42] Gething PW, Clara R. Burgert-Brucker. The DHS program modeled map surfaces: understanding the utility of spatial interpolation for generating indicators sub-national administrative levels. DHS Spatial Analysis Reports No. 15. Rockville: ICF; 2017.

[43] Osgood-Zimmerman A, Millear AI, Stubbs RW, Shields C, Pickering BV, Earl L, Graetz N, Kinyoki DK, Ray SE, Bhatt S, Browne AJ, Burstein R, Cameron E, Casey DC, Deshpande A, Fullman N, Gething PW, Gibson HS, Henry NJ, Herrero M, Krause LK, Letourneau ID, Levine AJ, Liu PY, Longbottom J, Mayala BK, Mosser JF, Noor AM, Pigott DM, Piwoz EG, Rao P, Rawat R, Reiner RC, Smith DL, Weiss DJ, Wiens KE, Mokdad AH, Lim SS, Murray CJL, Kassebaum NJ, Hay SI (2018). Mapping child growth failure in Africa between 2000 and 2015. Nature.

[44] Amegbor PM, Zhang Z, Dalgaard R, Sabel CE (2020). Multilevel and spatial analyses of childhood malnutrition in Uganda: examining individual and contextual factors. Sci Rep.

[45] Ugwu CLJ, Zewotir T (2020). Evaluating the effects of climate and environmental factors on Under-5 children malaria spatial distribution using generalized additive models (GAMs). J Epidemiol Global Health.

[46] Nelder JA, Wedderburn RW (1972). Generalized linear models. J Royal Stat Soc.

[47] Hastie TJ, Tibshirani RJ. Generalized additive models, vol 43. Boca Raton: CRC press; 1990.

[48] Wood SN. Generalized additive models: an introduction with R. Boca Raton: CRC press; 2017.

[49] Wand MP (2003). Smoothing and mixed models. Comput Stat.

[50] Wand H, Whitaker C, Ramjee G (2011). Geoadditive models to assess spatial variation of HIV infections among women in local communities of Durban, South Africa. Int J Health Geogr.

[51] Fahrmeir L, Kneib T (2009). Propriety of posteriors in structured additive regression models: theory and empirical evidence. J Stat Plan Inference.

[52] Gosoniu L, Vounatsou P, Sogoba N, Smith T (2006). Bayesian modelling of geostatistical malaria risk data. Geospat Health.

[53] Spiegelhalter DJ, Best NG, Carlin BP, van der Linde A (2002). Bayesian measures of model complexity and fit. J Royal Stat Soc.

[54] Bolstad WM. Understanding computational Bayesian statistics. 1st ed. New York: Wiley; 2009.

[55] Brezger A, Lang S (2006). Generalized structured additive regression based on Bayesian P-splines. Comput Stat Data Anal.

[56] Wand H, Whitaker C, Ramjee G (2011). Geoadditive models to assess spatial variation of HIV infections among women in local communities of Durban, South Africa. Int J Health Geogr.

[57] Rue H, Martino S, Chopin N (2009). Approximate Bayesian inference for latent Gaussian models by using integrated nested Laplace approximations. J Royal Stat Soc.

[58] Kelsall J, Wakefield J (1999). Discussion of ‘Bayesian models for spatially correlated disease and exposure data’, by Best et al. Bayesian Stat.

[59] Kazembe LN, Kandala N-B (2015). Estimating areas of common risk in low birth weight and infant mortality in Namibia: a joint spatial analysis at sub-regional level. Spatial Spatio-Temp Epidemiol.

[60] Lunn DJ, Thomas A, Best N, Spiegelhalter D (2000). WinBUGS-a Bayesian modelling framework: concepts, structure, and extensibility. Stat Comput.

[61] Wakefield J (2007). Disease mapping and spatial regression with count data. Biostatistics.

[62] Best NG (1999). Bayesian models for spatially correlated disease and exposure data. Bayesian Stat.

[63] Lang S, Brezger A (2000). Bayesx-software for bayesian inference based on markov chain Monte Carlo simulation techniques.

[64] Martins TG, Simpson D, Lindgren F, Rue H (2013). Bayesian computing with INLA: new features. Comput Stat Data Anal.

[65] Csa I (2016). Central statistical agency (CSA)[Ethiopia] and ICF.

[66] Demographic CE (2011). Health survey 2011.

[67] Macro O, bālaśelṭān, E.Y.s (2006). Demographic and Health Survey, 2005.

[68] Wamani H, Åstrøm AN, Peterson S, Tumwine JK, Tylleskär T (2007). Boys are more stunted than girls in sub-Saharan Africa: a meta-analysis of 16 demographic and health surveys. BMC Pediatr.

[69] Endris N, Asefa H, Dube L (2017). Prevalence of malnutrition and associated factors among children in rural Ethiopia. Biomed Res Int.

[70] Dasgupta A (2014). Assessment of under nutrition with composite index of anthropometric failure (CIAF) among under-five children in a rural area of West Bengal. Indian J Community Health.

[71] Akombi BJ, Agho KE, Merom D, Renzaho AM, Hall JJ (2017). Child malnutrition in sub-Saharan Africa: a meta-analysis of demographic and health surveys (2006-2016). PLoS One.

[72] Khamis AG (2020). The burden and correlates of childhood undernutrition in Tanzania according to composite index of anthropometric failure.

[73] Takele K, Zewotir T, Ndanguza D (2019). Understanding correlates of child stunting in Ethiopia using generalized linear mixed models. BMC Public Health.

[74] Kejo D, Mosha TCE, Petrucka P, Martin H, Kimanya ME (2018). Prevalence and predictors of undernutrition among underfive children in Arusha District, Tanzania. Food Sci Nutr.

[75] Farooq R, Khan H, Khan MA, Aslam M (2020). Socioeconomic and demographic factors determining the underweight prevalence among children under-five in Punjab. BMC Public Health.

[76] Ahmed MM (2016). Prevalence of undernutrition and risk factors of severe undernutrition among children admitted to Bugando medical Centre in Mwanza. Tanzania. BMC Nutr.

[77] Khamis AG, Mwanri AW, Kreppel K, Kwesigabo G (2020). The burden and correlates of childhood undernutrition in Tanzania according to composite index of anthropometric failure. BMC Nutr.

[78] Obasohan PE, Walters SJ, Jacques R, Khatab K (2020). Risk factors associated with malnutrition among children under-five years in sub-Saharan African countries: a scoping review. Int J Environ Res Public Health.

[79] World Health Organization. Indicators for assessing infant and young child feeding practices: definitions and measurement methods. Geneva: World Health Organization; 2021.

