# Supplementary material for: Spatial data analysis of malnutrition among children under-five years in Ethiopia
Source: BMC Med Res Methodol. 2021 Oct 27;21:232. doi: 10.1186/s12874-021-01391-x (PMC8549278; doi:10.1186/s12874-021-01391-x)
Supplement: Supplementary file 1 — Additional file 1. [file 12874_2021_1391_MOESM1_ESM.pdf]

```
###############################################################
###### Haile Mekonnen Fenta ######
################################################################
###############################################################
# GGAMM in R-INLA to identify the pattern and determinants of
#malnutrition among under-five age children   in Ethiopia ###
##############################################################
setwd("D:/PhDnurtion/papers/Paper2/finalpaper2/BMCRM_revision")##seting the working directory
### Activating INLA library and its dependence
#install.packages("INLA", repos=c(getOption("repos"), INLA="https://inla.r-inla-download.org/R/stable"), dep=TRUE)
# Packages required
library(rgdal)
library(splines)
library(sp)
library(maptools)
library(spdep)
library(INLA)
library(foreign)
library(shapefiles)
library(BayesX)
library(lattice)
library(R2BayesX)
library(sf)
inla.version()# we can see the INLA versions
## Importing the shape file
```

```
### Generating spatial weight matrix in R-InLA

shapef <- st_read("D:/PhDnurtion/papers/paper2/finalpaper2/BMCRM_revision/shepefile/ETH_adm2.shp")

temp <- poly2nb(shapef)

nb2INLA("Ethiopia.graph", temp)

Ethiopia_shape <- paste(getwd(),"/Ethiopia.graph",sep="")

#call the map

H=("D:/PhDnurtion/papers/paper2/finalpaper2/BMCRM_revision/Ethiopia.graph")

library(readr)

finaldata <- read_csv("D:/PhDnurtion/papers/paper2/finalpaper2/BMCRM_revision/finaldata.csv")

data=data.frame(finaldata)

data$CIAF=as.factor(data$CIAF)

attach(data)

data$CIAFRw[data$CIAF=="A"]=0

data$CIAFRw[data$CIAF=="CIAF"]=1

attach(data)

head(data)

####Potential models######

#### to add the weight, first we need to enable the weighted function in R inla

inla.setOption(enable.inla.argument.weights=TRUE)

GLMM<-inla(CIAFRw~factor(sex_child)+factor(breast_feeding)+factor(comorbidity) +factor(size_c)+ factor(DDS)+factor(type_birth)+
factor(place_R)+factor(edu_mother)+factor(edu_father)+factor(autonomy)+factor(work_mother)+factor(wateri)+factor(toileti)+factor(Ufive)+factor(media)+factor(wealth_i)+factor(DHS_year),family="binomial",data=data,control.compute=list(dic=TRUE,mlik=TRUE,cpo=TRUE,waic=TRUE),weights =weight)

GAMM<-inla(CIAFRw~factor(sex_child)+factor(breast_feeding)+factor(comorbidity) +factor(size_c)+ factor(DDS)+factor(type_birth)+
factor(place_R)+factor(edu_mother)+factor(edu_father)+factor(autonomy)+factor(work_mother)+facto
```

r(wateri)+factor(toileti)+factor(Ufive)+factor(media)+factor(wealth_i)+factor(DHS_year)+f(BMIMother)+f(bord)+f(HHsize)+

(aridity)+f(lst)+f(ur)+f(agechild)+f(evi),family="binomial",data=data,control.compute=list(dic=TRUE,mlik=TRUE,cpo=TRUE,waic=TRUE),verbose=TRUE,weights = weight)

including the spatial effect

data$zone.unstruct = data$zone

GGAMM<-inla(CIAFRw~factor(sex_child)+factor(breast_feeding)+factor(comorbidity) +factor(size_c)+factor(DDS)+factor(type_birth)+
factor(place_R)+factor(edu_mother)+factor(edu_father)+factor(autonomy)+factor(work_mother)+factor(wateri)+factor(toileti)+factor(Ufive)+factor(media)+factor(wealth_i)+factor(DHS_year)+f(BMIMother)+f(bord)+f(HHsize)+

f(aridity)+f(lst)+f(ur)+f(agechild)+f(evi)+ f(zone,model="besag",graph.file=H, param=c(1,0.01)) +

f(zone.unstruct,model="iid",param=c(1,0.01)),family="binomial",data=data,control.compute=list(dic=TRUE,mlik=TRUE,cpo=TRUE,waic=TRUE),verbose=TRUE,weights = weights)

####################generating both the fixed and random effects###

####prior sensitivity analysis for the selected model (GGAMM) #############

########## IG(a,b) IG(a,b) IG(a,b) IG(a,b)

#structured (0.001, 0.001) (0.01, 0.01) (0.05, 0.0005) (1, 0.026)

#unstructured (0.001, 0.001) (0.01, 0.01) (0.05, 0.0005) (1, 0.026)

###IG (0.001, 0.001)

GGAMM1<-inla(CIAFRw~factor(sex_child)+factor(breast_feeding)+factor(comorbidity) +factor(size_c)+factor(DDS)+factor(type_birth)+
factor(place_R)+factor(edu_mother)+factor(edu_father)+factor(autonomy)+factor(work_mother)+factor(wateri)+factor(toileti)+factor(Ufive)+factor(media)+factor(wealth_i)+factor(DHS_year)+f(BMIMother)+f(bord)+f(HHsize)+

f(aridity)+f(lst)+f(ur)+f(agechild)+f(evi)+ f(zone,model="besag",graph.file=H, param=c(0.001,0.001)) +

f(zone.unstruct,model="iid",param=c(0.001,0.001)),family="binomial",data=data,control.compute=list(dic=TRUE,mlik=TRUE,cpo=TRUE,waic=TRUE),verbose=TRUE,weights = weight)

##IG (0.01, 0.01)

GGAMM2<-inla(CIAFRw~factor(sex_child)+factor(breast_feeding)+factor(comorbidity) +factor(size_c)+ factor(DDS)+factor(type_birth)+ factor(place_R)+factor(edu_mother)+factor(edu_father)+factor(autonomy)+factor(work_mother)+factor(wateri)+factor(toileti)+factor(Ufive)+factor(media)+factor(wealth_i)+factor(DHS_year)+f(inla.group(BMIMother),model="rw2")+f(inla.group(bord),model="rw2")+f(inla.group(HHsize),model="rw2")+

f(inla.group(aridity),model="rw2")+f(inla.group(lst),model="rw2")+f(inla.group(ur),model="rw2")+f(inla.group(agechild),model="rw2")+f(inla.group(evi),model="rw2")+ f(zone,model="besag",graph.file=H, param=c(0.01,0.01)) +

f(zone.unstruct,model="iid",param=c(0.01,0.01)),family="binomial",data=data,control.compute=list(dic=TRUE,mlik=TRUE,cpo=TRUE,waic=TRUE),verbose=TRUE,weights = weight)

###IG (0.5, 0.0005)

GGAMM3<-inla(CIAFRw~factor(sex_child)+factor(breast_feeding)+factor(comorbidity) +factor(size_c)+ factor(DDS)+factor(type_birth)+ factor(place_R)+factor(edu_mother)+factor(edu_father)+factor(autonomy)+factor(work_mother)+factor(wateri)+factor(toileti)+factor(Ufive)+factor(media)+factor(wealth_i)+factor(DHS_year)+f(BMIMother)+ f(bord)+f(HHsize)+

f(aridity)+f(lst)+f(ur)+f(agechild)+f(evi)+ f(zone,model="besag",graph.file=H, param=c(0.05,0.0005)) +

f(zone.unstruct,model="iid",param=c(0.05,0.0005)),family="binomial",data=data,control.compute=list(dic=TRUE,mlik=TRUE,cpo=TRUE,waic=TRUE),verbose=TRUE,weights = weight)

###(1, 0.026)

GGAMM4<-inla(CIAFRw~factor(sex_child)+factor(breast_feeding)+factor(comorbidity) +factor(size_c)+ factor(DDS)+factor(type_birth)+ factor(place_R)+factor(edu_mother)+factor(edu_father)+factor(autonomy)+factor(work_mother)+factor(wateri)+factor(toileti)+factor(Ufive)+factor(media)+factor(wealth_i)+factor(DHS_year)+f(BMIMother)+ f(bord)+f(HHsize)+

```
f(aridity)+f(lst)+f(ur)+f(agechild)+f(evi)+ f(zone,model="besag",graph.file=H, param=c(1,0.026))
+

f(zone.unstruct,model="iid",param=c(1,0.026)),family="binomial",data=data,control.compute=list(dic=TRUE,mlik=TRUE,cpo=TRUE,waic=TRUE),verbose=TRUE,weight=weight)

summary(GGAMM1)

GGAMM1$dic

#####AUC computation for the given models#################

#install.packages(c("pROC","ROCit")) # Install the `pROC` and "ROCit" packages.

#summaries (mean, standard deviation and quantiles) of the fitted values

###a prediction (on the response scale) associated with each row of the input data

install.packages(c("pROC","ROCit"))

library(ROCit)

library(pROC)# Load the caret package into this R session.

pred_glmm=GLMM$summary.fitted.values$mean

pred_gamm=GAMM$summary.fitted.values$mean

pred_ggamm=GGAMM$summary.fitted.values$mean

Rocfinal=data.frame(pred_glmm,pred_gamm,pred_ggamm,CIAFw)

attach(Rocfinal)

####We can take the rounds

###Add this to code1

library(ROCit)
```

```
### for references, we will replace by y_test

roc1 <- plot.roc(as.vector(CIAFw),as.vector(pred_glmm), main="ROC comparison")

roc2 <- lines.roc(as.vector(CIAFw), as.vector(pred_gamm), percent=TRUE, col="blue")

roc3 <- rocit(as.vector(CIAFw),as.vector(pred_ggamm))

## Plot ROC curve

plot(roc1 , col = 1,legend = FALSE, YIndex = FALSE)

lines(roc2$TPR~roc2$FPR,col = 15, lwd = 0.5)

lines(roc3$TPR~roc3$FPR,col = 4, lwd = 0.5)

legend("bottomright", col = c(1,15,4),c("GLMM:AUC=0.5137",
"GAMM:AUC=0.5502","GGAMM:AUC=0.7218"), lwd = 0.5)

###Exact summary of the AUC

auc_glm = auc(roc_glmm)#Calculate the area under the ROC curve for GLMM

auc_gamm = auc(roc_gamm)#Calculate the area under the ROC curve for GAMM

auc_ggamm = auc(roc_ggamm)#Calculate the area under the ROC curve for GGAMM

#### summary statistics for AUC

auc_glm;auc_gamm;auc_ggamm#we can see the Area under the curve values
```
